# Computational Analysis of Artimisinin Derivatives on the Antitumor Activities

**DOI:** 10.1007/s13659-017-0142-x

**Published:** 2017-11-01

**Authors:** Hui Liu, Xingyong Liu, Li Zhang

**Affiliations:** School of Chemical Engineering, Sichuan University of Science & Engineering, Zigong, China

**Keywords:** QSAR, CoMFA, CoMSIA, Topomer CoMFA, HQSAR, Artemisinin

## Abstract

The study on antitumor activities of artemisinin and its derivatives has been closely focused on in recent years. Herein, 2D and 3D QSAR analysis was performed on the basis of a series of artemisinin derivatives with known bioactivities against the non-small-cell lung adenocarcinoma A549 cells. Four QSAR models were successfully established by CoMSIA, CoMFA, topomer CoMFA and HQSAR approaches with respective characteristic values q^2^ = 0.567, R^2^ = 0.968, ONC = 5; q^2^ = 0.547, R^2^ = 0.980, ONC = 7; q^2^ = 0.559, R^2^ = 0.921, ONC = 7 and q^2^ = 0.527, R^2^ = 0.921, ONC = 6. The predictive ability of CoMSIA with r^2^ = 0.991 is the best one compared with the other three approaches, such as CoMFA (r^2^ = 0.787), topomer CoMFA (r^2^ = 0.819) and HQSAR (r^2^ = 0.743). The final QSAR models can provide guidance in structural modification of artemisinin derivatives to improve their anticancer activities.

## Introduction

Cancer ranks the top public health threat and is the main cause of death in China [1]. In 2015, more than 4 million new cancer cases and nearly three million deaths occurred in China, in which lung cancer is the most common incident cancer and it is also the highest mortality cancer. Although a series of chemical therapies are available, many of which would accompany with serious side effects due to the chemical toxicity to normal cells. Additionally, drug resistance during the cancer treatment is of great challenge to be overcame. These challenges urged researchers to develop novel antitumor drugs.

Natural products are treasure in the process of drug discovery. Artemisinin (ART), a sesquiterpene lactone natural product, isolated from the traditional Chinese medicinal herb Artemisia annua L., is a powerful antimalarial drug. In 1991, Scholars reported that artemisinin derivatives showed inhibitory activity to leukemia P388 cell [2]. Afterward, Henry’s group revealed that dihydroartemisinin combined with holotransferrin exhibited powerful inhibitory activity against leukemia cell line and breast cancer cells with minor effects on normal human lymphocytes [3, 4]. In the past decades, increasing publications have been disclosed in the area of the artemisinin and its derivatives as antitumor compounds [5–7].

Over the years, Artemisinin was widely used as antimalarial drug and potential anticancer lead compound, but the clear target and mechanism are still unknown. Furthermore, there are some pharmacokinetic limitations of artemisinin, such as low solubility in water or organic media, low bioavailability and short plasma half-life in vivo [8]. To overcome these problems, a series of artemisinin derivatives such as artemether, Artemisia ether, dihydroartemisinin, artesunate were designed to improve both antimalarial and anticancer activities [9].

Quantitative structure activity relationship (QSAR)which encompasses several analysis methods such as comparative molecular field analysis (CoMFA), comparative molecular similarity indices analysis (CoMSIA) [10, 11], topomerCoMFA [12] and hologram QSAR (HQSAR) [13] was widely used in drug development process. Numerous researches documenting QSAR studies on the antimalarial artemisinin have been published. Cheng and co-workers established CoMFA and CoMSIA models to study artemisinin and its analogues as antimalarial agents. They concluded that the CoMFA and CoMSIA models had a good predictive ability and well matched the docking results [10]. Avery group reported the CoMFA and HQSAR analysis on a series of 211 artemisinin derivatives with known antimalarial activity [14]. Yadavand co-workers employed QSAR and molecular docking to screen the novel antimalarial artemisinin derivatives [15]. By contrast, there are few QSAR studies on the anticancer property of artemisinin. In this work, traditional QSAR, topomer QSAR and HQSAR based on 46 compounds against A549 cell were performed to get useful information to guide the structural modification of artemisinin for improvement of its antitumor activities.

## Experimental Section

### Data Sets

An array of artemisinin derivatives in Table 1 with reported anticancer activities against the non-small-cell lung adenocarcinoma A549 cells were selected [16, 17]. The molecular structures were prepared by D.S 4.0 (Discovery Studio 4.0 Client). Those 3D structures were optimized by energy minimization with SYBYL- × 2.0. The IC_50_ values in units were converted in logarithmic unit (pIC_50_). The relationships between the chemical structures and pIC_50_ against A549 were listed in Table 1. During the development of QSAR models, the rational selection of training and test sets have an impact on the predictive ability of the established model [18]. The marked nine compounds by * in Table 1 were selected as the test set.

**Table 1:** Structures and corresponding pIC_50_ values of artemisinin derivatives

Compounds	Ar	Y	X	pIC_50_
**1**	–	–	–	4.100
**2**	C_6_H_5_	–	–	5.911
**3**	2–Br–C_6_H_4_	–	–	6.130
**4**	3–Br–C_6_H_4_	–	–	6.359
**5**	4–Br–C_6_H_4_	–	–	7.327
**6**	3,4–(OMe)_2_–C_6_H_3_	–	–	6.655
**7**	3–C_6_H_4_O–C_6_H_4_–CF_3_	–	–	6.764
**8**	C_6_H_5_	–	–	6.179
**9**	2–F–C_6_H_4_	–	–	6.438
**10**	4–F–C_6_H_4_	–	–	6.514
**11**	2–Cl–C_6_H_4_	–	–	4.974
**12**	2–Br–C_6_H_4_	–	–	6.714
13*	3–Br–C_6_H_4_	–	–	7.070
**14**	4–Br–C_6_H_4_	–	–	7.408
**15**	3–C_6_H_4_O–C_6_H_4_–CF_3_	–	–	6.853
**16**	C_6_H_5_	(CH_2_)_2_	O	5.619
**17**	2––Cl–C_6_H_4_	(CH_2_)_2_	O	6.073
**18**	2–Br–C_6_H_4_	(CH_2_)_2_	O	6.303
**19**	4–CN–C_6_H_4_	(CH_2_)_2_	O	5.146
20*	4–OMe–C_6_H_4_	(CH_2_)_2_	O	5.199
**21**	2,4–(OMe)_2_–C_6_H_3_	(CH_2_)_2_	O	5.146
**22**	3,4–(OMe)_2_–C_6_H_3_	(CH_2_)_2_	O	5.083
**23**	3–OMe,4–Obz–C_6_H_3_	(CH_2_)_2_	O	5.313
**24**	C_6_H_5_	(CH_2_)_5_	O	5.334
25*	C_6_H_5_	(CH_2_)_2_	NH	5.327
**26**	2–F–C_6_H_4_	(CH_2_)_2_	NH	5.366
27*	3–F–C_6_H_4_	(CH_2_)_2_	NH	5.327
**28**	4–F–C_6_H_4_	(CH_2_)_2_	NH	5.337
29*	3–Cl–C_6_H_4_	(CH_2_)_2_	NH	5.522
**30**	4–Cl–C_6_H_4_	(CH_2_)_2_	NH	5.366
**31**	2–Br–C_6_H_4_	(CH_2_)_2_	NH	5.337
32*	3–Br–C_6_H_4_	(CH_2_)_2_	NH	5.318
**33**	4–Br–C_6_H_4_	(CH_2_)_2_	NH	5.376
**34**	4–NMe_2_–C_6_H_4_	(CH_2_)_2_	NH	5.356
**35**	2–naphthyl	(CH_2_)_2_	NH	5.275
**36**	C_6_H_5_	(CH_2_)_2_	NCH_3_	4.891
**37**	2–F–C_6_H_4_	(CH_2_)_2_	NCH_3_	4.854
38*	3–F–C_6_H_4_	(CH_2_)_2_	NCH_3_	4.872
39*	2–Cl––C_6_H_4_	(CH_2_)_2_	NCH_3_	5.214
**40**	4–Cl–C_6_H_4_	(CH_2_)_2_	NCH_3_	5.134
**41**	2–Br–C_6_H_4_	(CH_2_)_2_	NCH_3_	4.793
**42**	4–Br–C_6_H_4_	(CH_2_)_2_	NCH_3_	5.275
**43**	2,4–Me2–C_6_H_3_	(CH_2_)_2_	NCH_3_	5.115
**44**	3(3–CF_3_C_6_H_4_)OC_6_H_4_	(CH_2_)_2_	NCH_3_	5.602
**45**	C_6_H_5_	1,2–C_6_H_4_	NCH_3_	4.962
46*	C_6_H_5_	CH = CH(cis)	NCH_3_	4.716

*Molecules used in the test set

### Structure Preparation and Alignments

**Fig. 1:**
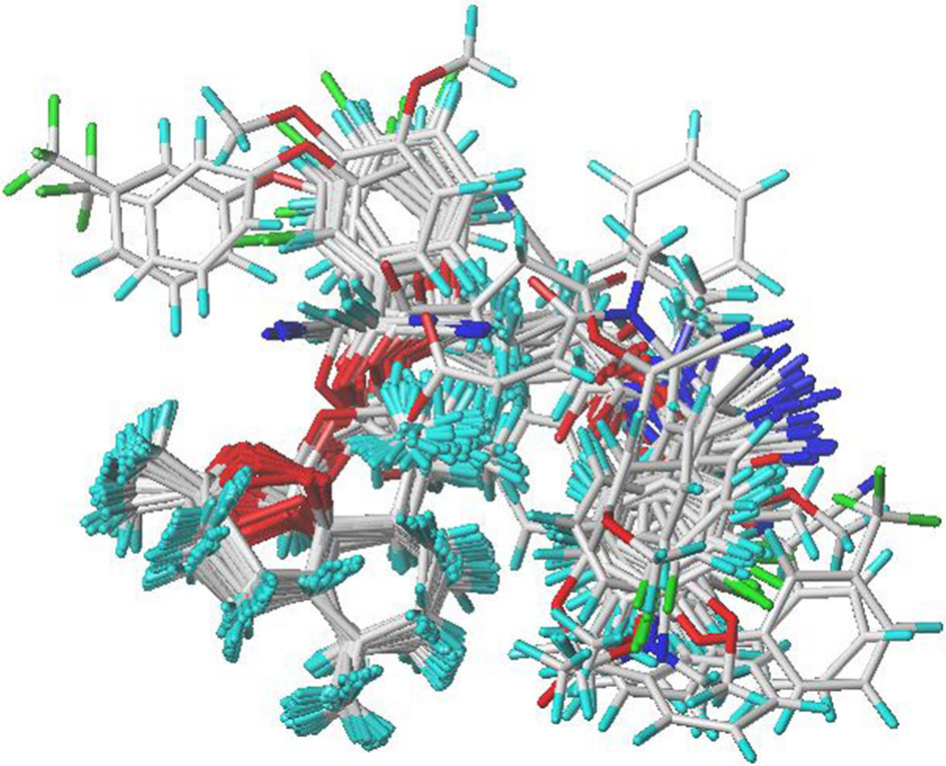
The alignment of 46 artemisinin derivatives

The results of CoMFA and CoMSIA analysis depend on the molecular alignment [19]. It is vital to choose a template compound at this stage [20]. The geometry optimizations of the ligands were performed by Powell conjugated gradient algorithm method using the TPIPOS force field until the root-mean-square (RMS) deviation reached 0.005 kcal/mol. The Gasteiger Hückel approach was applied to calculate partial atomic charges. In this study, a ligand-based alignment was adopted since the receptor is unknown. Compound 14, the most potent inhibitor among those selected ligands, was chosen as the template which all molecules were aligned based on. The alignment of structures was shown in Fig. 1.

### CoMFA and CoMSIA Methodology

In CoMFA, the energies parameters of steric and electrostatic of the CoMFA were set by the default probe, i.e., a *sp*3 carbon atom and + 1 charge as steric and electrostatic probe respectively. And Tripos force field with a distance-dependent dielectric constant at all intersections with a fixed grid spacing of 2 Å was used. The maximum steric and electrostatic energy thresholds were set to be 30 kcal/mol. Firstly, the partial least squares (PLS) analysis was performed based on the CoMFA descriptors as independent variables and pIC_50_ values as dependent variables. The leave-one-out (LOO) cross-validation method was used in regression analysis. The column filtering was set to be 3 kcal/mol to improve the signal–noise ratio by omitting those lattices points which energy variation was below this threshold. Then the cross-validation was performed to obtain the optimum number of components (ONC), standard error of predictions (SEP) and cross-validation squared correlation coefficient (q^2^). The optimum number of components (ONC) was used for the non-cross validation PLS analysis to build the final model with the corresponding conventional correlation coefficient (r^2^), standard error of estimate (SEE)and the F value.

Five CoMSIA fields including steric, electrostatic, hydrophobic, hydrogen bond donor and hydrogen bond acceptor have been evaluated using the default probe, charge, and grid spacing which were similar to the CoMFA model. The column filtering was set to be 3 kcal/mol and the attenuation factor was set to be 0.3. The subsequent parameters were obtained in the same way as described above in CoMFA.

### TopomerCoMFA

TopomerCoMFA, an objective QSAR methodology, accelerates lead optimization [12]. Being different from the traditional CoMFA, topomerCoMFA is a No-Alignment CoMFA [21]. In topomerCoMFA, electrostatic and steric fields were calculated for the fragments from 3D structural cleavage.

### Hologram Quantitative Structure–Activity Relationship

Compared with the traditional 2D fingerprint, HQSAR encodes more information including branched and cyclic fragments as well as stereochemistry. The hologram was constructed by the HQSAR descriptors which encoded topological and compositional molecular information [22]. In general, the best HQSAR model was obtained by various hologram lengths and the fragment.

In order to instruct the predictive ability of QSAR models, the predictive correlation coefficient (r^2^) was calculated using the following equation based on the test set:

$${\text{r}}^{2}\, { = }\,\frac{{{\text{SD}} - {\text{PRESS}}}}{\text{SD}}$$

In this equation, SD indicates the sum of square of the difference between the true values of the test set and the mean data of the training set; PRESS is the sum of square of the difference between the predicted and the observed activities of the test set.

## Results and Discussion

In this work, 37 artemisinin derivatives were randomly selected as a training set to generate QSAR models. The rest of molecules were used to validate the model as a test set. For an ideal model, the following values with regard to statistics parameters should be required. The predictive correlation coefficient r^2^ of the test set must be greater than 0.50 and the cross-validation squared correlation coefficient q^2^ of the training set must better than 0.5. In addition, Pearson correlation coefficient R^2^ values and standard error of estimate (SEE) as well as the former quantity vary in the range 0–1, of which 1 represents a perfect model and 0 means a model without any predictive ability. The optimum results are listed in Table 2. The statistics parameters q^2^, R^2^ (training set) and r^2^ (test set) are 0.567, 0.968 and 0.991 respectively, which indicated that the CoMSIA represented the best model.

**Table 2:** Summary of the statistical results of CoMFA, CoMSIA, topomerCoMFA and HQSAQ models

Method	CoMFA	CoMSIA	Topomer CoMFA	HQSAR
Optimum components	7	5	7	6
q^2^	0.547	0.567	0.559	0.527
SEP	0.579	0.547	0.57	0.581
R^2^ (training set)	0.980	0.968	0.921	0.921
SEE (training set)	0.122	0.148	0.24	0.238
r^2^ (test set)	0.787	0.991	0.819	0.743
F-ratio	199.966	189.930	–	–
Best length	–	–	–	151
Intercept	–	–	4.83	–
Field contribution	Steric = 0.472 Electrostatic = 0.528	Steric = 0.095 Electrostatic = 0.363 Hydrophobic = 0.312 Acceptor = 0.23	–	–

PLS analysis was performed using the training and test sets with pIC_50_ values. Debugging the column filtering of 3 led to q^2^ = 0.547 and optimum number of components = 7. The statistics parameters of CoMFA model were listed in Table 2, in which a high R^2^ (0.98) and a low SEE (0.122) were obtained from the training set and an estimated F-ratio value of 199.966. In the model, the steric and electrostatic fields contributed 47.2 and 52.8% respectively. These data showed that the CoMFA model is reasonable.

A series of models were established by random combination of five fields such as electrostatic (E), steric (S), hydrophobic (H), hydrogen bond acceptor (A) and hydrogen bond donor (D). Among these models, four fields (S/E/H/A) were selected to establish the best CoMSIA model. The statistics results in Table 2 showed that the cross-validation correlation coefficient q^2^ = 0.567, optimum number of components = 5, and the SEP = 0.547. The non-cross-validated coefficient R^2^ of 0.968 with a low SEE of 0.148 was obtained. The contributions of S, E, H and A were 9.5, 36.3, 31.2 and 23% respectively. These values indicate that a reliable CoMSIA model was constructed successfully.

**Fig. 2:**
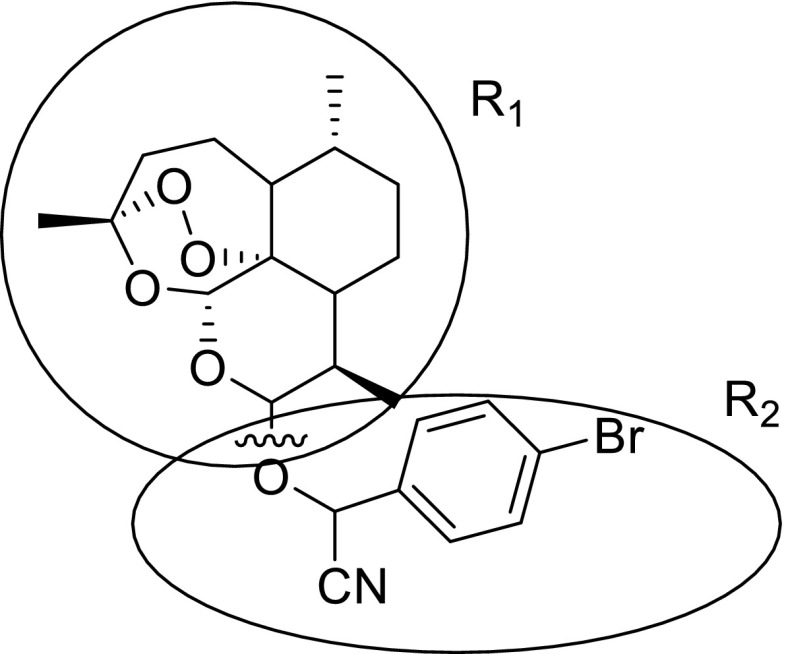
Splitting mode was used in topomerCoMFA

In topomerCoMFA, the first step is to split molecules into fragments from the rotatable bonds. The best data was obtained by splitting the C-O bonds at the C10 of training and test sets, as shown in Fig. 2 when compound 14 was chosen as template. Test set was selected in the same way as described in CoMFA and CoMSIA models. The statistics results were listed in Table 2 with q^2^ = 0.559 when the optimum number of components was set to be 7 and R^2^ = 0.921 with a low SEE of 0.24. The r^2^ of 0.819 for test set was obtained by the equation as mentioned above. The corresponding predictive activity of the training and test sets and contribution values of each fragment were exhibited in Table 3.

**Table 3:** Experimental and predicted data of all compounds using topomerCoMFA with contribution values of each fragment

Compounds	pIC_50_ (exp.)	pIC_50_ (pre.)	Res.	Contribution of R_2_
**1**	4.1	4.0847	0.0153	− 0.7422
**2**	5.9111	6.156	− 0.2449	1.3291
**3**	6.1301	5.9517	0.1784	1.1248
**4**	6.3595	6.3803	− 0.0208	1.5534
**5**	7.3279	7.4108	− 0.0829	2.5839
**6**	6.6556	6.5735	0.0821	1.7466
**7**	6.7645	6.7876	− 0.0231	1.9607
**8**	6.1791	6.156	0.0231	1.3291
**9**	6.4389	6.3058	0.1331	1.4789
**10**	6.514	6.5291	− 0.0151	1.7022
**11**	4.9746	5.896	− 0.9214	1.0691
**12**	6.7144	5.9517	0.7627	1.1248
**13**	7.0705	6.3803	0.6902	1.6066
**14**	7.4089	7.4108	− 0.0019	2.5839
**15**	6.8538	6.7876	0.0662	1.9607
**16**	5.6197	5.53	0.0897	0.7031
**17**	6.0731	6.1108	− 0.0377	1.2839
**18**	6.3036	6.1568	0.1468	1.3299
**19**	5.1469	5.0149	0.132	0.188
**20**	5.1992	5.5107	− 0.3115	0.6838
**21**	5.1466	5.2192	− 0.0726	0.3923
**22**	5.083	5.0012	0.0818	0.1743
**23**	5.313	5.3631	− 0.0501	0.5362
**24**	5.334	5.2812	0.0528	0.4544
**25**	5.3279	5.3531	− 0.0252	0.5262
**26**	5.3665	5.3472	0.0193	0.5203
**27**	5.3279	5.2399	0.088	0.413
**28**	5.337	5.4063	− 0.0693	0.5794
**29**	5.5228	5.2696	0.2532	0.4427
**30**	5.3665	5.4498	− 0.0833	0.6229
**31**	5.337	5.3434	− 0.0064	0.5165
**32**	5.3187	5.2446	0.0741	0.4177
**33**	5.3767	5.4738	− 0.0971	0.6469
**34**	5.3565	5.4058	− 0.0493	0.5789
**35**	5.2757	5.2608	0.0149	0.4339
**36**	4.8913	4.9147	− 0.0234	0.0878
**37**	4.8548	4.8832	− 0.0284	0.0563
**38**	4.8728	4.8325	0.0403	0.0056
**39**	5.2146	4.9323	0.2823	0.1054
**40**	5.134	5.1027	0.0313	0.2758
**41**	4.793	4.9289	− 0.1359	0.102
**42**	5.2757	5.1671	0.1086	0.3402
**43**	5.1157	5.1318	− 0.0161	0.3049
**44**	5.602	5.5867	0.0153	0.7598
**45**	4.9625	4.9362	0.0263	0.1093
**46**	4.7167	4.9712	− 0.2545	0.1443

The training and test sets were selected in the same means for CoMFA, CoMSIA and topomerCoMFA models. QSAR analysis was performed by screening the hologram lengths of 97, 151, 199, 257, 307 and 353, using the fragment size of 5–8 to select the best model based on the least standard error. The best results were summarized in Table 2, in which a cross-validated q^2^ = 0.527 with 6 optimal components and a hologram length of 151 were obtained. The conventional R^2^ value and standard error of estimate were 0.921, 0.238 respectively.

### Validation of the QSAR Models

**Fig. 3:**
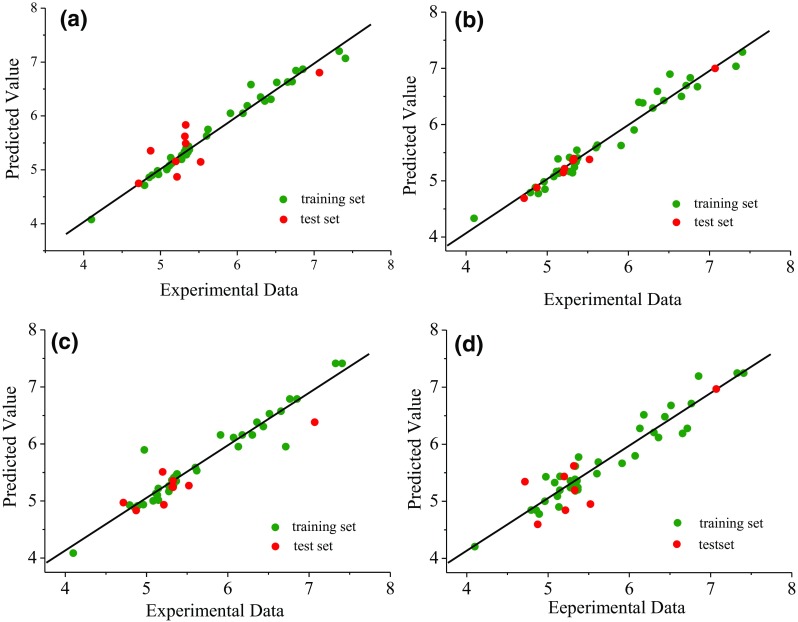
Scatter diagram analysis using experimental and predicted data of QSAR models. **a** CoMFA; **b** CoMSIA; **c** topomerCoMFA; **d** HQSAR

The statistics results of the best models for these four QSAR methods were collected in Table 2. The experimental data, prediction values and its residues of the training set of the CoMFA, CoMSIA and HQSAR models were summarized in Table 4, and the rest of 9 molecules in the test set were used to verify the predictive capacity of these models as shown in Table 5. The relevant data of the best topomerCoMFA was exhibited in Table 3. From the Table 2, rational r^2^ values were obtained to validate the reliability and predictability of the CoMFA, CoMSIA, HQSAR and topomerCoMFA models (0.787, 0.991, 0.743 and 0.819 respectively). The Fig. 3 graphically explained the correlation of predicted and experimental data.

**Table 4:** Experimental dates, prediction values and its residues of the training set used for the CoMFA, CoMSIA and HQSAR

Compounds	pIC_50_	CoMFA		CoMSIA		HQSAR	
		Pred.	Res.	Pred.	Res.	Pred.	Res.
**1**	4.1	4.078	0.022	4.331	− 0.231	4.204	− − 0.104
**2**	5.9111	6.049	− 0.1379	5.627	0.2841	5.664	0.2471
**3**	6.1301	6.188	− 0.0579	6.395	− 0.2649	6.276	− 0.1459
**4**	6.3595	6.276	0.0835	6.591	− 0.2315	6.116	0.2435
**5**	7.3279	7.202	0.1259	7.036	0.2919	7.246	0.0819
**6**	6.6556	6.629	0.0266	6.501	0.1546	6.187	0.4686
**7**	6.7645	6.841	− 0.0765	6.83	− 0.0655	6.711	0.0535
**8**	6.1791	6.58	− 0.4009	6.383	− 0.2039	6.514	− 0.3349
**9**	6.4389	6.309	0.1299	6.426	0.0129	6.481	− 0.0421
**10**	6.514	6.621	− 0.107	6.896	− 0.382	6.679	− 0.165
**11**	4.9746	4.915	0.0596	4.848	0.1266	5.429	− 0.4544
**12**	6.7144	6.637	0.0774	6.693	0.0214	6.276	0.4384
**14**	7.4089	7.066	0.3429	7.289	0.1199	7.246	0.1629
**15**	6.8538	6.866	− 0.0122	6.671	0.1828	7.192	− 0.3382
**16**	5.6197	5.749	− 0.1293	5.633	− 0.0133	5.685	− 0.0653
**17**	6.0731	6.05	0.0231	5.902	0.1711	5.795	0.2781
**18**	6.3036	6.35	− 0.0464	6.293	0.0106	6.204	0.0996
**19**	5.1469	5.118	0.0289	5.17	− 0.0231	5.435	− 0.2881
**21**	5.1466	5.122	0.0246	5.133	0.0136	5.193	− 0.0464
**22**	5.083	5.005	0.078	5.075	0.008	5.326	− 0.243
**23**	5.313	5.335	− 0.022	5.141	0.172	5.26	0.053
**24**	5.334	5.402	− 0.068	5.329	0.005	5.383	− 0.049
**26**	5.3665	5.387	− 0.0205	5.543	− 0.1765	5.199	0.1675
**28**	5.337	5.368	− 0.031	5.312	0.025	5.181	0.156
**30**	5.3665	5.436	− 0.0695	5.382	− 0.0155	5.244	0.1225
**31**	5.337	5.281	0.056	5.24	0.097	5.614	− 0.277
**33**	5.3767	5.373	0.0037	5.405	− 0.0283	5.773	− 0.3963
**34**	5.3565	5.327	0.0295	5.333	0.0235	5.357	− 0.0005
**35**	5.2757	5.265	0.0107	5.414	− 0.1383	5.238	0.0377
**36**	4.8913	4.898	− 0.0067	4.771	0.1203	4.777	0.1143
**37**	4.8548	4.858	− 0.0032	4.88	− 0.0252	4.844	0.0108
**40**	5.134	5.225	− 0.091	5.387	− 0.253	4.9	0.234
**41**	4.793	4.713	0.08	4.787	0.006	4.845	− 0.052
**42**	5.2757	5.194	0.0817	5.162	0.1137	5.357	− 0.0813
**43**	5.1157	5.075	0.0407	5.164	− 0.0483	5.087	0.0287
**44**	5.602	5.628	− 0.026	5.587	0.015	5.484	0.118
**45**	4.9625	4.98	− 0.0175	4.982	− 0.0195	5	− 0.0375

**Table 5:** Experimental dates, prediction values and its residues of the test set used for the CoMFA, CoMSIA and HQSAR

Compounds	pIC_50_	CoMFA		CoMSIA		HQSAR	
		Pred.	Res.	Pred.	Res.	Pred.	Res.
**13**	7.0705	6.803	0.2675	6.998	0.0725	6.967	0.1035
**20**	5.1992	5.157	0.0422	5.146	0.0532	5.433	− 0.2338
**25**	5.3279	5.831	− 0.5031	5.371	− 0.0431	5.192	0.1359
**27**	5.3279	5.49	− 0.1621	5.394	− 0.0661	5.197	0.1309
**29**	5.5228	5.147	0.3758	5.378	0.1448	4.949	0.5738
**32**	5.3187	5.62	− 0.3013	5.358	− 0.0393	5.619	− 0.3003
**38**	4.8728	5.354	− 0.4812	4.876	− 0.0032	4.596	0.2768
**39**	5.2146	4.87	0.3446	5.215	− 0.0004	4.843	0.3716
**46**	4.7167	4.748	− 0.0313	4.689	0.0277	5.343	− 0.6263

These four QSAR models showed that there were good linear relationships between the true values and predicted values for the group of the training set and the test set (Fig. 3). In comparison, the dispersity of the HQSAR is distinctly higher than the other QSAR models, indicating that 3D QSAR methods (CoMFA, CoMSIA, and topomerCoMFA) are more suitable to provide crucial structural modificative information than 2D QSAR (HQSAR). Meanwhile, the CoMSIA model has better predictive ability than CoMFA and topomerCoMFA models observed from the Table 2 and the Fig. 3. It is assumed that four fields (A/H/S/E) from the CoMSIA model are more comprehensive than two fields (S/E) from CoMFA and topomerCoMFA models.

### Graphical Analysis of QSAR Models

**Fig. 4:**
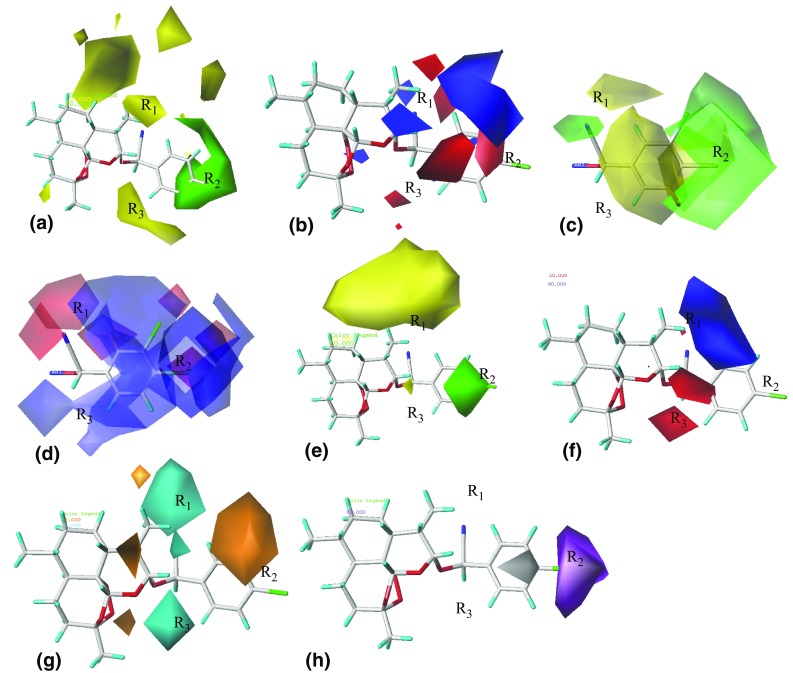
3D QSAR models contour plots in combination with compound **14** as the template. **a** CoMFA steric field; **b** CoMFA electrostatic field; **c** TopomerCoMFA steric field; **d** TopomerCoMFA electrostatic field; **e** CoMSIA steric field; **f** CoMSIA electrostatic field; **g** CoMSIA hydrogen bond acceptor field; **h** CoMSIA hydrophobic field

In this work, contour maps were calculated using the PLS analysis (StDev * Coeff) and the contour plots of 3D QSAR models with compound 14 as the template are shown in Fig. 4. In this section, the molecular skeleton was labeled as R_1_, R_2_, and R_3_ regions for the sake of contrastive analysis.

In the steric contour maps, the green contours (80% contributions) represent that bulk group is a favorable substituent and the yellow contours (20% contributions) show that bulk group is an unfavorable substituent at this position. In the CoMFA model, a big green polyhedron at the R_2_ portion means that big groups are useful to improve the activities, which matches the case that when the groups like Br, methoxyl replace the hydrogen atom in this position, compounds 3, 4, 5, 6, 7 have a higher activity than compound 2, and compound 15 is higher than compound 8. Two yellow contours appear in the map, one of which is near the R_3_ region and the other is above the C–9 methyl. The steric contour maps of the CoMSIA (Fig. 4e) and the topomerCoMFA (Fig. 4c) models are similar with the CoMFA steric map.

Similarly, in the electrostatic contour maps, the blue contours (80% contributions) means that the positive charged groups are good for the active values while the red contours (20% contributions) indicate that positive charged groups would decrease the activity in this region. In the CoMFA model, a large blue contour near the region of R_2_ indicates that the positive charged groups in this position are favorable to the activity. Some red contours appearing at the R_1_ and R_3_ suggest that the negative charged groups were useful to increase the inhibitory activity in this area. It can be explained that fluorinated and bromine substituent compounds 3 and 9, 12 have a better activity than non-substituted compounds 2 and 8. The electrostatic field distribution of the CoMSIA and the topomerCoMFA models are also similar with the CoMFA as shown in Fig. 4d, f.

Particularly, in the CoMSIA model, the hydrogen bond acceptor and hydrophobic contour maps are exhibited in Fig. 4g, h. The favored and disfavored hydrogen bond acceptor is represented using cyan (80% contributions) and orange (20% contributions) polyhedrons. There are two cyan polyhedrons near R_1_, R_3_ and an orange contour at the region of R_2_ as shown in Fig. 4g. On the other hand, in the hydrophobic contour maps, the violet regions (80% contributions) means that hydrophobic groups are favorable for improving activity in this place, where as white regions (20% contributions) indicate that hydrophobic groups are disfavored. There is a large violet polyhedron near the R_2_ of the compound 14, and it means that the hydrophobic groups in this area are benefic for increasing the bioactivity. It is exampled by Compounds 6, 7 and 15 with alkoxy substituent showing good bioactivity in dataset. There is also a disfavored white contour around the benzene ring which means that hydrophilic groups improve the bioactivity in this region.

**Fig. 5:**
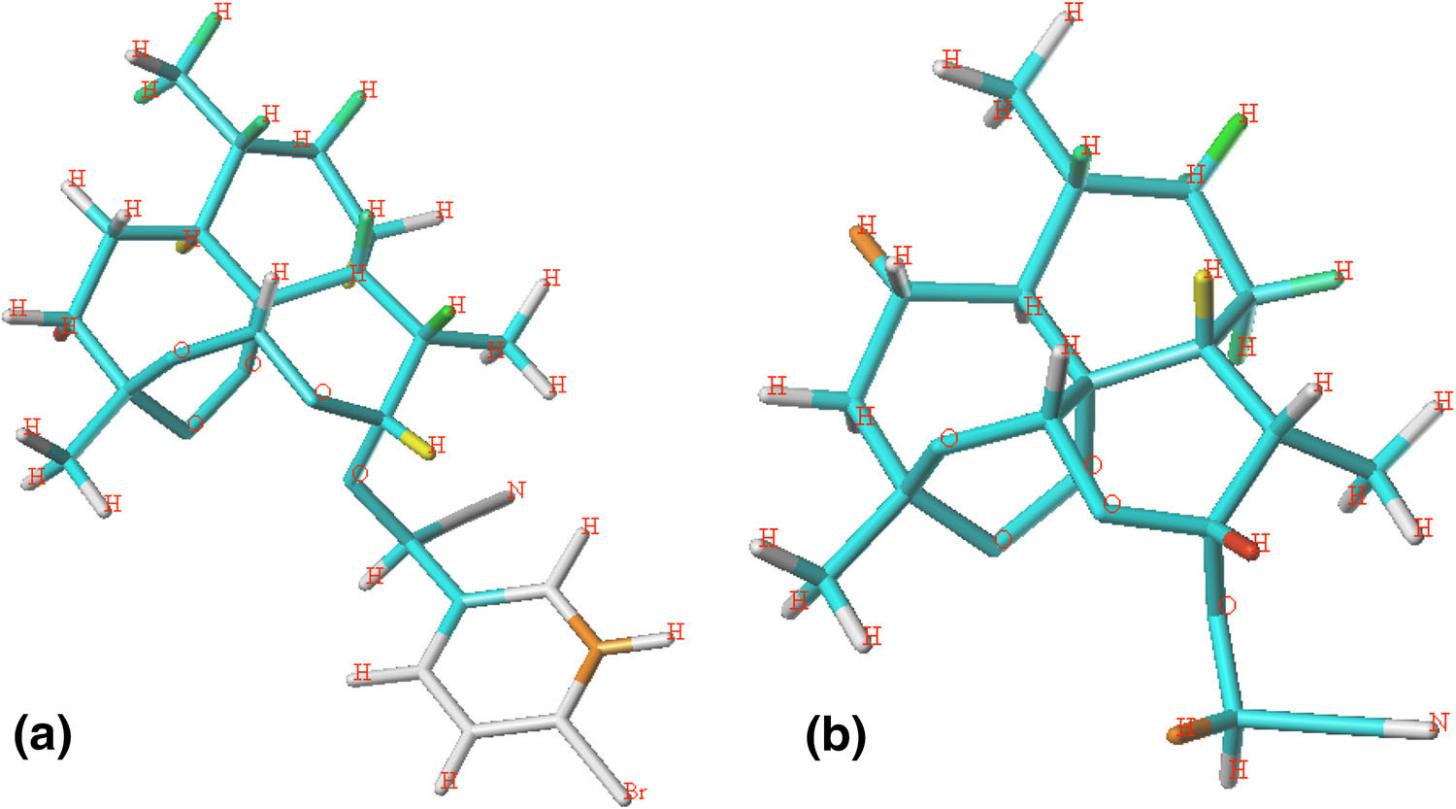
Contribution maps from HQSAR for compounds **14** (**a**) and **1** (**b**)

The HQSAR model was performed to reach two major objectives listed as following: (a) accurate prediction of the activities of untested compounds; (b) visual display of the contributions of fragments to the activity of the compounds. The atomic contribution maps which display the individual atomic contributions to the molecule’s activity by the color of atoms are shown in Fig. 5. Red, red orange and orange atoms represent negative contribution, while the white atoms represent medium contributions to the model. Yellow, green blue and blue exhibit positive contribution. The most and least active molecules as the template were shown as compound 14 and 1 respectively in the contribution maps. The common structural fragments of artemisinin skeleton are displayed by green blue which contribute positively to the model (Fig. 5).

### Mode to Optimize Structures

**Fig. 6:**
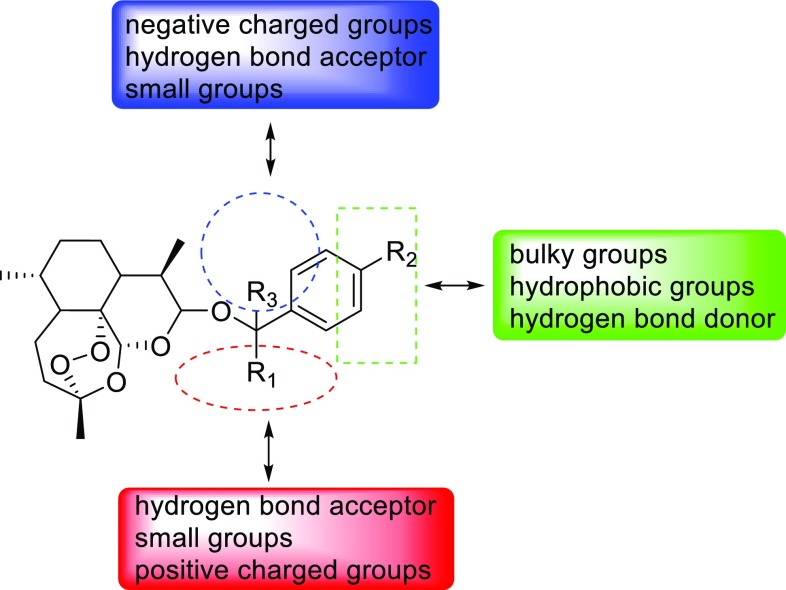
The summary of structural modification for artemisinin derivatives from the QSAR models

Based on the results of QSAR models, the mode to optimize structures with compound 14 as the template was summarized in Fig. 6. As illustrated in the oval region, the small groups, positive charged groups and hydrogen bond acceptor are favorable for improving activity, while using the bulky hydrophobic groups or hydrogen bond donor at the rectangle place is beneficial to promote the bioactivity of the molecules. In addition, it can improve the molecular bioactivity if using the negative charged groups, small groups or hydrogen bond acceptor at the circular region.

## Conclusion

In this study, Four QSAR models (CoMFA, CoMSIA, topomerCoMFA, HQSAR) were successfully established based on a series of artemisinin derivatives with known bioactivity data, which show promising predictive ability validated by the test set. We found that the 3D QSAR models are more suitable than 2D QSAR. Structure–activity relationship information (A/H/S/E) were established from the CoMSIA model with a higher predictive ability (r^2^ = 0.991) than CoMFA and topomerCoMFA as well as HQSAR. The topomerCoMFA showed better results than CoMFA and also take much less time. In addition, the 2D atomic contribution maps from HQSAR showed the contribution of individual atoms to the activity of each molecule. Thus, this study will provide reasonable suggestions for design of new artemisinin derivatives with potent antitumor activities.

## References

[1] Chen W, Zheng R, Baade PD, Zhang S, Zeng H, Bray F, Jemal A, Yu XQ, He J (2016). CA Cancer J. Clin..

[2] Deng DA, Cai JC (1991). Youji Huaxue.

[3] Lai H, Singh NP (1995). Cancer Lett..

[4] Singh NP, Lai H (2001). Life Sci..

[5] Binh LH, Van NTT, Kien VT, My NTT, Van Chinh L, Nga NT, Tien HX, Thao DT, Vu TK (2016). Med. Chem. Res..

[6] Xu CC, Deng T, Fan ML, Lv WB, Liu JH, Yu BY (2016). Eur. J. Med. Chem..

[7] Blazquez AG, Fernandez-Dolon M, Sanchez-Vicente L, Maestre AD, Gomez-San Miguel AB, Alvarez M, Serrano MA, Jansen H, Efferth T, Marin JJ, Romero MR (2013). Bioorg. Med. Chem..

[8] Crespo-Ortiz MP, Wei MQ (2012). J. Biomed. Biotechnol..

[9] Posner GH, Park SB, Gonzalez L, Wang D, Cumming JN, Klinedinst D, Shapiro TA, Bachi MD (1996). J. Am. Chem. Soc..

[10] Cheng F, Shen J, Luo X, Zhu W, Gu J, Ji R, Jiang H, Chen K (2002). Bioorg. Med. Chem..

[11] Saeed ME, Kadioglu O, Seo EJ, Greten HJ, Brenk R, Efferth T (2015). Anticancer Res..

[12] Cramer RD (2003). J. Med. Chem..

[13] Tong W, Lowis DR, Perkins R, Chen Y, Welsh WJ, Goddette DW, Heritage TW, Sheehan DM (1998). J. Chem. Inf. Comput. Sci..

[14] Avery MA, Alvim-Gaston M, Rodrigues CR, Barreiro EJ, Cohen FE, Sabnis YA, Woolfrey JR (2002). J. Med. Chem..

[15] Yadav DK, Dhawan S, Chauhan A, Qidwai T, Sharma P, Bhakuni RS, Dhawan OP, Khan F (2014). Curr. Drug Targets.

[16] Li Y, Wu J-M, Shan F, Wu G-S, Ding J, Xiao D, Han J-X, Atassi G, Leonce S, Caignard D-H, Renard P (2003). Biorg. Med. Chem..

[17] Wu JM, Shan F, Wu GS, Li Y, Ding J, Xiao D, Han JX, Atassi G, Leonce S, Caignard DH, Renard P (2001). Eur. J. Med. Chem..

[18] Roy K (2007). Exp. Opin Drug Discov..

[19] Gaurav A, Singh R (2012). Med. Chem. (Shariqah (United Arab Emirates)).

[20] Lalit M, Gangwal RP, Dhoke GV, Damre MV, Khandelwal K, Sangamwar AT (2013). J. Mol. Struct..

[21] Heidari A, Fatemi MH (2017). Chem. Biol. Drug Des..

[22] Myint KZ, Xie XQ (2010). Int. J. Mol. Sci..

